# Patient Outcomes and Nurse Understaffing in For-Profit Hospitals

**DOI:** 10.1097/MLR.0000000000002376

**Published:** 2026-09-03

**Authors:** Matthew D. McHugh, Karen B. Lasater, K. Jane Muir, Angelo Petto, Trissa Lyman, Linda H. Aiken

**Affiliations:** * Center for Health Outcomes and Policy Research, University of Pennsylvania School of Nursing, Philadelphia, PA; † Leonard Davis Institute for Health Economics, University of Pennsylvania, Philadelphia, PA

**Keywords:** hospital ownership, for-profit, nurse staffing, patient outcomes, causal mediation

## Abstract

**Background:**

For-profit hospital ownership has been associated with worse patient outcomes, but the mechanisms remain unclear. Underinvestment in nurse staffing is a plausible pathway linking for-profit ownership to performance.

**Objectives:**

To examine whether differences in nurse staffing mediate associations between for-profit hospital ownership and patient, nurse, and hospital outcomes.

**Design:**

Cross-sectional study using 2024 data on hospital ownership, registered nurse survey responses, Medicare claims, and Hospital Compare measures.

**Subjects:**

A total of 143 for-profit and 798 nonprofit adult, nonfederal, acute care hospitals across 10 US states; >1.17 million Medicare fee-for-service admissions; and 17,368 direct-care bedside registered nurses.

**Measures:**

Patient-level 30-day mortality and readmission (Medicare Provider Analysis and Review); hospital-level hybrid risk-standardized mortality and readmission and HCAHPS overall hospital rating (CMS Hospital Compare); nurse burnout (Maslach Burnout Inventory emotional exhaustion subscale) and related outcomes (Penn Nurses All survey). The hypothesized mediator was hospital-level nurse staffing.

**Results:**

Among surgical patients, for-profit ownership was associated with a 0.48 percentage-point higher 30-day mortality (95% CI: 0.24–0.73), 45% mediated through staffing. Among medical patients, 1.13 percentage-point higher 30-day mortality (95% CI: 0.52–1.73), 40% mediated. Readmission findings were similar. For-profit status was associated with worse HCAHPS ratings (−4.45 percentage points; 95% CI: −5.20 to −3.60), 31% mediated. Nurse burnout was 9 percentage points higher (95% CI: 5.8–12.4), 67% mediated.

**Conclusions:**

For-profit hospital ownership is associated with worse patient and nurse outcomes, and differences in nurse staffing account for a share of these disparities.

In seminal work in the early 1980s, sociologist Paul Starr warned of inevitable tensions between profit-seeking and patient care, as corporate interests, not clinicians and patients, increasingly set the terms of care.^1^ This warning has become increasingly salient as health care corporatization has accelerated in recent years,^2,3^ with over a third of US hospitals owned by for-profit entities.^4^ Several firms are seeing record stock market performance, underscoring the growing influence of investor priorities in the health care sector.^5,6^ A substantial, though not entirely consistent, evidence base links for-profit hospital ownership to worse patient outcomes, including higher mortality and readmissions, more potentially avoidable care/procedures (eg, cesarean section), and reduced access to essential care.^7–14^ Similar patterns have been documented among for-profit nursing homes,^15,16^ hospice,^17,18^ and dialysis facilities.^19,20^

Underinvestment in nursing is a plausible pathway from for-profit ownership to worse outcomes (for both patients and nurses), given the potential to reduce labor costs by investing less in nurse staffing and high-quality work environments, and the centrality of nursing to hospital performance. Yet, relatively few studies have directly evaluated whether and how nursing resources differ between for-profit and nonprofit hospitals, and whether these differences help explain observed differences in outcomes.^9,21^ Our study evaluates how nurse staffing levels differ in for-profit hospitals compared with nonprofit hospitals, whether those disparities account in part for nurse and patient outcomes differences between the 2 types of hospitals, and whether differences in nurse staffing represent a policy-relevant mechanism linking hospital ownership to performance.

## METHODS

### Study Design

This cross-sectional study used data on nurse staffing levels and patient, nurse, and hospital outcomes in nonprofit (excluding public) and for-profit acute care hospitals from a survey of registered nurses (RNs) across 10 states (2024 Penn Nurses) merged with patient outcomes data from 2024 Medicare Provider Analysis and Review (MedPAR) claims and 2024 Hospital Compare data. The study was approved by the [University of Pennsylvania] Institutional Review Board, with a waiver of informed consent, for the analysis of deidentified secondary data. The parent study producing the nurse survey data was approved by the same IRB, and participation was voluntary.

### Population and Data

Our analysis focused on comparing patient and nurse outcomes in adult, nonfederal, acute care for-profit (proprietary/investor-owned) versus nonprofit hospitals. We used a variety of sources to confirm ownership, including 4 files from the Centers for Medicare & Medicaid Services (CMS): hospital-level enrollments file; hospital-owner-level file; a hospital-addresses-level file; and a file of hospitals with multiple national provider identifiers. We cross-checked these with data from the American Hospital Association (AHA) annual survey, and in cases of discrepancies, referenced PitchBook, hospital websites, and, where available, state licensure files. We also used AHA data for hospital structural characteristics such as bed size, teaching status, and rural location. Operating margins (overall operating margin and patient-care operating earnings), payer-mix and safety-net measures (Medicare DSH index, Medicaid inpatient day share, Medicare inpatient day share, non-Medicare/non-Medicaid inpatient day share, and uncompensated care share), and Medicare case-mix index were assessed using RAND Hospital Data derived from HCRIS files.^22^ In addition, we characterized hospital market conditions using the Herfindahl-Hirschman Index (HHI), a measure of hospital market concentration at the hospital referral region level, where higher values indicate greater market concentration and less competition.

Patient-level data and outcomes (ie, 30 d mortality and readmissions) were derived from MedPAR claims files that include detailed patient-level data, including admitting diagnosis/procedure, comorbid conditions, demographics, and relevant outcomes. For comparability across hospitals, we restricted our attention to common surgical (general, orthopedic, and vascular) and medical (congestive heart failure, acute myocardial infarction, pneumonia, stroke, and sepsis) patients. Hospital-level quality outcomes were measured using CMS Hospital Compare hybrid risk-standardized all-cause mortality and unplanned 30-day readmission rates, which integrate claims and electronic health record data to adjust for patient case mix and enable cross-hospital comparisons. We also used Hospital Compare’s Hospital Consumer Assessment of Healthcare Providers and Systems (HCAHPS) data on patient satisfaction ratings of hospital care.

Nurse outcomes and our measure of nurse staffing came from the 2024 [Penn Nurses] survey of nurses who serve as informants about the conditions of their employment settings. Invitations to participate in the survey were emailed to 100% of actively licensed RNs in 9 states (CA, FL, NJ, OR, WA, NM, NY, IL, LA) and mailed to a random sample of RNs licensed in PA, where email addresses were not available. We followed a modified Dillman survey approach, and nonrespondents received follow-up reminders at regular intervals. The survey was conducted between December 2023 and March 2024 and yielded over 113,000 responses. The large survey sampling frame reflects the fact that although many licensed RNs are providing direct patient care in hospital settings, the licensure lists as a sampling frame do not include information on place of employment. Thus, respondents identify their place of employment by selecting their hospital from a list of licensed facilities within their state. Research has shown^23^ through a gold standard double-sampling technique that involves intensive nonresponder surveying, that our approach yields little evidence of nonresponse bias at the nurse level and allows us to eliminate response bias at the hospital level, the most critical level for representation. Our analysis focused on respondents who identified as RNs employed as direct-care (bedside) staff nurses in hospitals on inpatient units.

### Variables

#### Nurse Staffing

We assessed nurse staffing levels by aggregating nurses’ responses to an item asking direct care staff nurses to indicate their level of agreement on the degree to which their assigned patient workload is safe. When aggregated, this measure ranges from 0 to 4 and varies considerably across hospitals; it has the attractive feature of incorporating more than a numeric ratio, which may not capture all aspects of workload, and enables meaningful comparisons across all hospital settings. The hospital-aggregated measure demonstrated adequate aggregate reliability [intraclass correlation coefficient, ICC(2)=0.74]. As a test of sensitivity, we also conducted analyses using the staffing and resource adequacy subscale of the Practice Environment Scale of the Nursing Work Index (PES-NWI), an instrument endorsed by the Partnership for Quality Measurement (PQM) (formerly National Quality Forum) as an organization-level nurse staffing performance measure.^24,25^ Prior research supports these nurse-reported assessments as a valid measure of staffing adequacy.^26^ Bedside nurses are uniquely positioned to evaluate staffing in their work settings because they directly experience the match between available staff and patient needs. Metrics such as nursing hours-per-patient-day provide standardized counts, but can miss the real-time, contextual dimensions of nursing workload, including acuity fluctuations, unit-level resource availability, and care complexity that nurses’ judgments naturally incorporate.^27^

#### Patient Outcomes

Thirty-day mortality is our primary patient outcome and was defined as a death occurring within 30 days of hospital admission and included out-of-hospital deaths. Thirty-day readmission included patients readmitted to any hospital within 30 days after being discharged alive to home or home with home health care. We excluded those who died in the hospital, were admitted to hospice, or were transferred to other facilities. Hospital-wide mortality and readmission were measured using CMS Hospital Compare hybrid outcome measures, which estimate risk-standardized all-cause mortality and unplanned 30-day readmission across multiple conditions. We also evaluated hospital-level patient experience based on the overall rating of the hospital (% of patients rating a 9 or 10 out of 10). Patient-level covariates included age, sex, transfer-in status, emergent admission, condition- or procedure-specific mortality/readmission risk estimated by a model fit to an external data set, and comorbidity burden based on 30 comorbidities. Risk adjustment was enhanced using a 180-day lookback to capture comorbidities reported in previous hospitalizations. For surgical patients, the models incorporated dummy variables based on DRG code, indicating the specific surgical procedure. The risk adjustment for medical conditions included previously validated covariates specific to each condition (eg, pneumonia, acute myocardial infarction).

#### Nurse Outcomes

We assessed a series of individual-level nurse outcomes derived from survey data including our primary nurse outcome of burnout (via the emotional exhaustion subscale of the Maslach Burnout Inventory),^28^ nurses’ job dissatisfaction; intention to leave the job in the next year; quality of care on the unit was fair or poor rather than good or excellent; and lack of confidence that hospital management would resolve patient care problems. Nurses also gave their unit an overall grade on patient safety and were also asked whether they would recommend their hospital as either a place of care or employment. Nurse-level covariates included age, sex, educational attainment (bachelor’s degree or higher), tenure at the employing institution, and full- versus part-time employment status.

### Data Analysis

We conducted causal mediation analyses^29^ within a potential outcomes framework using the mediation package in R to examine whether hospital nurse staffing mediated the association between for-profit hospital ownership and patient, nurse, and hospital outcomes. For each individual-level patient outcome, 2 models were estimated. First, the mediator was modeled at the hospital level using linear regression; that is, the staffing mediator was regressed on hospital for-profit ownership and hospital-level covariates, including bed size category, teaching status, rural location, hospital market concentration [Herfindahl-Hirschman Index (HHI)], Medicare case-mix index, and Medicare disproportionate share hospital (DSH) index. Second, the outcome model estimated the association between the mediator and the binary outcome (eg, 30 d mortality) using a multilevel logistic regression with a random intercept for hospital to account for clustering of patients within hospitals. The outcome model included hospital for-profit ownership, the staffing mediator, and patient- and hospital-level covariates as outlined above, such as age, sex, condition- or procedure-specific mortality/readmission risk, comorbidities, and hospital characteristics. Using the fitted mediator and outcome models, we estimated the average causal mediation effect (ACME), representing the indirect effect of for-profit ownership on mortality operating through the mediator (staffing); the average direct effect (ADE), representing the effect of for-profit ownership not operating through the mediator; the total effect; and the proportion of the total effect mediated. Because the outcome model was nonlinear and included random effects, mediation effects were estimated using simulation-based inference. Confidence intervals were obtained using 1000 Monte Carlo simulations. Effects were reported as average marginal effects on the probability scale. We estimated a comparable set of models for nurse outcomes, but using individual nurse characteristics (eg, age, education level, full-time/part-time status) instead of patient characteristics. The Hospital Compare outcomes were at the hospital level, so the series of models were all hospital-level and involved linear outcomes. We conducted formal sensitivity analyses using the medsens procedure in R^29^ to quantify how strong an unobserved confounder of the mediator-outcome relationship would need to be to attenuate the estimated average causal mediation effect (ACME) to 0. The procedure parameterizes the residual correlation between the error terms of the mediator model and the outcome model as ρ, then computes the critical value of ρ at which the ACME would equal 0. Larger absolute values of the critical ρ indicate greater robustness to unmeasured mediator-outcome confounding.

## RESULTS

Table 1 shows that, compared with nonprofit hospitals, for-profit hospitals were less likely to be large or major teaching institutions and were less often located in rural areas. For-profit hospitals had substantially higher operating margins and patient care operating margins, whereas hospital market concentration, measured by the HHI, was similar across ownership groups. Payer and financial-burden measures were mixed: nonprofit hospitals had somewhat higher Medicaid inpatient day share and uncompensated care share; however, only the difference in uncompensated care share reached statistical significance. Medicare inpatient day share, non-Medicare/Medicaid inpatient day share, and Medicare case-mix index did not differ significantly. Despite these similarities in several structural and payer-mix characteristics, for-profit hospitals had worse nurse staffing, with lower mean safe staffing scores (2.0 in for-profit vs. 2.4 in nonprofit hospitals), which corresponds with 71% of for-profit nurses reporting that their staffing levels were not safe, compared with 54% in nonprofit hospitals. Staffing and resource adequacy scores were also lower in for-profit hospitals (1.8 vs. 2.3). For-profit hospitals had higher rates of nurse burnout compared with nonprofits (53% vs. 44%, respectively). They also had more intent to leave, job dissatisfaction, low confidence in management’s ability to resolve safety concerns, poorer quality of care, and a lower likelihood of recommending the hospital as either a place of care or employment. For-profit hospitals had worse patient outcomes, including higher risk-standardized mortality and readmission rates, and lower overall hospital ratings from patients.

**TABLE 1:** Characteristics of Hospitals by For-Profit Status

Characteristic	Nonprofit (n=798), n (%)	For-profit (n=143), n (%)	*P*
No. beds			0.004
≤100	176 (22)	28 (20)	
101–250	276 (35)	70 (49)	
>250	346 (43)	45 (32)	
Teaching status			0.017
Nonteaching	340 (43)	76 (53)	
Minor teaching	289 (36)	50 (35)	
Major teaching	169 (21)	17 (12)	
Rural location	118 (14.8)	8 (5.6)	0.005
Operating margin (%), mean±SD	−1.8±10.9	6.9±12.8	<0.001
Patient care operating margin (%), mean±SD	11.5±12.1	16.9±13.7	<0.001
Herfindahl-Hirschman Index, mean±SD	1804±1505	1763±1487	0.765
Medicaid inpatient day share (%), mean±SD	26.5±13.1	24.4±13.2	0.083
Medicare inpatient day share (%), mean±SD	27.2±11.8	25.4±9.3	0.085
Non-Medicare/Medicaid inpatient day share (%), mean±SD	67.0±12.0	68.5±8.8	0.169
Uncompensated care share (%), mean±SD	9.0±5.7	7.0±7.1	0.002
Medicare case-mix index, mean±SD	1.81±0.30	1.78±0.20	0.220
Safe staffing, mean±SD	2.4±0.4	2.0±0.3	<0.001
Staffing and resource adequacy, mean±SD	2.3±0.4	1.8±0.3	<0.001
% of nurses reporting outcomes, mean±SD			
Burnout	43.9±12.7	52.5±14.2	<0.001
Intent to leave	21.0±10.2	35.5±14.2	<0.001
Job dissatisfaction	29.7±11.8	41.2±13.6	<0.001
Not confident that management will resolve safety issues	65.1±15.7	81.4±10.9	<0.001
Quality of care rated as poor or fair	24.6±14.7	41.9±16.6	<0.001
Would not recommend the hospital to family	29.3±17.7	54.3±18.4	<0.001
Would not recommend as a workplace	28.1±15.4	52.2±17.6	<0.001
Patient outcomes, mean±SD			
Hybrid hospital risk-standardized mortality rate	4.0±0.5	4.2±0.5	<0.001
Hybrid hospital risk-standardized readmission rate	15.0±0.8	15.7±0.9	<0.001
Overall hospital rating score	87.1±3.3	83.0±4.2	<0.001

Table 2 shows that patients in for-profit and nonprofit hospitals were similar in age and sex distribution, with comparable proportions of medical and surgical admissions overall. Comorbidity burden was not clinically meaningfully different across ownership types. Despite broadly similar patient characteristics, outcomes were worse in for-profit hospitals, with higher 30-day mortality (9.9% vs. 8.8%) and readmission rates (13.7% vs. 12.2%) compared with nonprofit hospitals.

**TABLE 2:** Patient Characteristics and Outcomes by For-Profit Status

Characteristic	Nonprofit (n=1,026,966), n (%)	For-profit (n=144,548), n (%)	*P*
Age (y), mean±SD	78.1±8.5	78.1±8.4	0.267
Sex: male	468,786 (45.6)	65,750 (45.5)	0.250
Medical admissions	618,632 (60.2)	87,919 (60.8)	<0.001
Diagnosis-related group			<0.001
Pneumonia	85,697 (8.3)	13,344 (9.2)	
Stroke	66,476 (6.5)	9684 (6.7)	
Acute myocardial infarction	40,200 (3.9)	6857 (4.7)	
Congestive heart failure	141,751 (13.8)	17,974 (12.4)	
Sepsis	284,508 (27.7)	40,060 (27.7)	
Surgical admissions	408,334 (39.8)	56,629 (39.2)	<0.001
Surgery type			<0.001
General surgery	118,475 (11.5)	15,102 (10.4)	
Orthopedic surgery	243,579 (23.7)	34,116 (23.6)	
Vascular surgery	46,280 (4.5)	7411 (5.1)	
Comorbidity index (mortality)	10.36 (15.7)	10.27 (15.4)	0.033
Comorbidity index (readmission)	10.10 (8.6)	9.39 (8.3)	<0.001
Outcomes			
30 d mortality	90,635 (8.8)	14,249 (9.9)	<0.001
30 d readmission	116,784 (12.2)	18,462 (13.7)	<0.001

Nurses in for-profit hospitals were slightly older, with an average age of close to 48 years old, but had fewer years of experience at their current hospital and were less likely to hold a BSN or higher degree (Table 3). Nurses in for-profit hospitals were more likely to report high burnout (55%) than nurses in nonprofits (44%). Across all other measured outcomes, nurses in for-profit hospitals reported substantially worse conditions, including greater intent to leave, higher job dissatisfaction, lower confidence in management resolving care issues, poorer care quality, and a lower likelihood of recommending the hospital as a place of care or employment.

**TABLE 3:** Nurse Characteristics and Outcomes by For-Profit Status

Characteristic	Nonprofit (n=16,142), n (%)	For-profit (n=1226), n (%)	*P*
Age (y), mean±SD	46.7 (12.5)	47.8 (12.5)	0.005
Years at current hospital, mean±SD	11.1 (11.0)	8.8 (10.2)	<0.001
Female	14,084 (87.3)	1057 (86.2)	0.296
Bachelor of Science in Nursing or higher	12,477 (77.3)	788 (64.3)	<0.001
Full-time employment	12,323 (76.3)	997 (81.3)	<0.001
Nurse-reported outcomes			
Burnout	7036 (44.1)	663 (54.7)	<0.001
Intent to leave	3130 (19.4)	395 (32.3)	<0.001
Job dissatisfaction	4689 (29.1)	515 (42.0)	<0.001
Not confident that management will resolve safety issues	10,340 (64.8)	989 (81.3)	<0.001
Quality of care rated as poor or fair	3691 (23.1)	504 (41.4)	<0.001
Would not recommend the hospital to family	4177 (26.1)	622 (51.1)	<0.001
Would not recommend as a workplace	4277 (26.5)	649 (53.0)	<0.001

Among surgical patients, for-profit ownership was associated with a 0.48 percentage-point (95% CI: 0.24–0.73) increase in 30-day mortality risk, of which 45% [average causal mediation effect (ACME) 0.22 percentage points; 95% CI: 0.13–0.32] was mediated through staffing differences (Table 4). For surgical readmissions, the total effect was 1.76 percentage points, with 30% (0.54 percentage points; 95% CI: 0.35–0.74) mediated through staffing. Among medical patients, for-profit ownership was associated with a 1.13 percentage-point (95% CI: 0.52–1.73) increase in 30-day mortality risk, with 0.45 percentage points (40%) mediated through staffing (ACME; 95% CI: 0.25–0.68). For medical readmissions, the total effect was 2.04 percentage points (95% CI: 1.54–2.53), with 16% (0.34 percentage points) being mediated through less safe staffing in for-profits.

**TABLE 4:** Causal Mediation Effects of Nurse Staffing in the Association Between For-Profit Hospital Ownership and Patient Outcomes

Outcome	Total effect, estimate	Total effect, 95% CI	ACME, estimate	ACME, 95% CI	Mediated (%)
Surgical patients					
30 d mortality	0.48	(0.24–0.73)	0.22	(0.13–0.32)	45
30 d readmission	1.76	(1.27–2.25)	0.54	(0.35–0.74)	30
Medical patients					
30 d mortality	1.13	(0.52–1.73)	0.45	(0.25–0.68)	40
30 d readmission	2.04	(1.54–2.53)	0.34	(0.18–0.53)	16
Hospital-level outcomes					
Hybrid mortality	0.23	(0.12–0.34)	0.11	(0.07–0.15)	46
Hybrid readmission	0.58	(0.42–0.76)	0.12	(0.07–0.19)	21
Overall hospital rating	−4.45	(−5.20 to −3.60)	−1.38	(−1.75 to −1.03)	31

Effects are reported as absolute differences in outcome probability for patient-level binary outcomes expressed in percentage points and as absolute differences in risk-standardized rates for hospital-level outcomes.

ACME indicates the average causal mediation effect.

Results were similar when using hospital-level hybrid risk-standardized all-cause mortality and unplanned 30-day readmission rates (Table 4). For-profit status was associated with higher hybrid risk-standardized mortality (total effect=0.23; 95% CI: 0.12–0.34), with 46% of the association mediated through the indirect pathway (ACME=0.11; 95% CI: 0.07–0.15) of workload. For the hybrid 30-day readmission risk-standardized rate, the total for-profit effect was 0.58 (95% CI: 0.42–0.76) with 21% mediated (ACME=0.12; 95% CI: 0.07–0.19). We found that for-profit status was also associated with worse overall hospital ratings on HCAHPS (total effect=−4.45; 95% CI: −5.20 to −3.60), with approximately one-third of the effect mediated (31%; ACME=−1.38; 95% CI: −1.75 to −1.03) by lower safe staffing in for-profit institutions.

Table 5 shows that across nurse outcomes, staffing demonstrated large and consistent mediated pathways, accounting for approximately one-third to two-thirds of total effects, suggesting that less safe staffing levels in for-profit hospitals were an intermediate mechanism for the worse nurse outcomes observed in for-profit hospitals. Burnout was 9 percentage points higher in for-profits (total effect; 95% CI: 5.8–12.4), with 67% of the effect mediated (ACME=6 percentage points; 95% CI: 4.5–7.7) by less safe staffing. Findings were similar across all other outcomes. The intent to leave was 12 percentage points (95% CI: 8.5–15.0) higher, with 36% mediated by staffing. Job dissatisfaction and low confidence in management were 12 and 15 percentage points higher, respectively, with 58% and 53% of effects mediated. Nurses in for-profits were more likely to report poor or fair quality of care (17 percentage points higher; 65% mediated) and unwillingness to recommend the hospital to family (22 percentage points higher; 51% mediated) or as a workplace (25 percentage points higher; 47% mediated).

**TABLE 5:** Causal Mediation Effects of Nurse Staffing in the Association Between For-Profit Hospital Ownership and Nurse Outcomes

Outcome	Total effect, estimate	Total effect, 95% CI	ACME, estimate	ACME, 95% CI	Mediated (%)
Burnout	9.1	(5.8–12.4)	6.1	(4.5–7.7)	67
Intent to leave	11.6	(8.5–15.0)	4.2	(3.0–5.5)	36
Job dissatisfaction	11.6	(8.3–15.1)	6.7	(5.0–8.7)	58
Low confidence in management	14.8	(11.7–17.7)	7.9	(6.2–9.7)	53
Quality of care rated as poor or fair	16.5	(12.8–20.8)	10.7	(8.1–13.0)	65
Would not recommend the hospital to family	22.3	(17.7–27.0)	11.3	(8.5–14.4)	51
Would not recommend as a workplace	25.2	(20.8–29.4)	11.7	(8.9–14.5)	47

Effects are reported as absolute differences in outcome probability expressed in percentage points for nurse-level binary outcomes.

ACME indicates the average causal mediation effect.

Sensitivity analyses (Supplemental Table 1, SDC1, Supplemental Digital Content 1, https://links.lww.com/MLR/D242) indicate that the mediated effects were generally robust to unmeasured mediator-outcome confounding. For 6 of the 7 nurse outcomes and for the HCAHPS overall hospital rating, the average causal mediation effect would be nullified only under substantial residual mediator-outcome correlation (critical |ρ| ≈ 0.4–0.6). For intent to leave, sensitivity was moderate (|ρ| ≈ 0.3). The mortality and readmission mediation findings were more sensitive to unmeasured confounding (|ρ| ≈ 0.3 and 0.2, respectively), consistent with their smaller absolute effect sizes. We also conducted our analysis replacing the staffing measure with the Staffing and Resource Adequacy subscale of the PES-NWI and observed qualitatively similar results (Supplemental Tables 2 and 3, SDC1, Supplemental Digital Content 1, https://links.lww.com/MLR/D242).

## DISCUSSION

We found consistent evidence that for-profit hospital ownership is associated with worse patient, nurse, and hospital-level outcomes, and that differences in nurse staffing account for a portion of these disparities. Nurses in for-profit hospitals reported less safe staffing, higher burnout, greater job dissatisfaction, and stronger intentions to leave, alongside worse patient outcomes, including higher risk-adjusted mortality and readmission rates and lower patient experience ratings. In causal mediation analyses, safe nurse staffing accounted for 16% to 46% of the association between for-profit ownership and patient mortality and readmissions, and for approximately one-third to two-thirds of the associations with adverse nurse outcomes. For example, for-profit ownership was associated with a 0.5-percentage-point higher 30-day mortality risk, approximately 5 additional deaths per 1000 admissions, which was partly mediated by differences in nurse staffing. These findings are consistent with a hypothesized mechanism linking for-profit ownership to outcomes through lower investment in nursing resources, though cross-sectional mediation analysis cannot establish this pathway definitively.

These results have direct policy relevance amid accelerating hospital corporatization and expanding for-profit ownership. In some states, more than one-third of hospitals are for-profit, yet few regulatory guardrails exist to address potential quality and safety concerns. Public awareness of ownership-related risks remains limited,^30^ and transparency regarding nurse staffing levels is minimal.^31^ Hospitals have historically been regarded as essential community institutions, but deteriorating safety and working conditions may undermine public trust and make some hospitals less attractive to both patients and clinicians, echoing patterns previously observed in largely for-profit nursing homes.^32,33^ Policies establishing minimum safe nurse staffing standards could serve as an important safeguard to protect patients and stabilize the hospital nursing workforce.^34,35^

State and federal efforts to strengthen staffing standards,^36^ enhance ownership transparency,^37^ and incorporate workforce measures into payment and accountability programs^38^ may meaningfully improve patient, nurse, and hospital outcomes. Treating safe nurse staffing as a core component of health care quality rather than a discretionary operating expense may be particularly important in for-profit settings, where incentives to constrain labor costs may be strongest.

Limitations that warrant consideration include the cross-sectional design, which limits causal inference regarding ownership. However, the mediation framework, extensive covariate adjustment, and sensitivity analyses strengthen the plausibility of the proposed pathway. Although we adjusted for a range of patient- and hospital-level characteristics, we could not directly adjust for all hospital- and market-level factors that may confound the ownership-outcome and ownership-staffing relationships, physical infrastructure, physician composition and employment arrangements, postacute care referral networks, leadership quality, and local hospital-market conditions. Prior studies using alternative identification strategies have reported a range of estimates for the effect of for-profit hospital ownership on patient outcomes. Instrumental-variable approaches have found small or null effects on inpatient outcomes,^39^ and work using hospital ownership conversions in a difference-in-differences framework has similarly reported limited changes in mortality following conversion.^13^ Our larger cross-sectional associations likely reflect stable between-hospital differences in organizational and workforce practices that conversion-based designs (ie, testing whether changing ownership changes performance within a hospital) are not designed to detect. Our contribution is therefore less about identifying the average causal effect of ownership per se and more about quantifying how much of the observed performance gap between for-profit and nonprofit hospitals is consistent with a pathway operating through the specific, policy-actionable input of nurse staffing. Although unmeasured confounding cannot be fully excluded, our sensitivity analyses indicated that most mediated effects would be nullified only under moderate-to-strong residual confounding (critical |ρ| ≈ 0.3–0.6); sensitivity was weakest for the smallest mediated effects (hybrid readmission), although a growing body of literature suggests nurse staffing is associated with readmission.^35,40,41^ In addition, our staffing measure reflects nurses’ direct appraisals of whether their assigned patient workloads were safe. Results were materially unchanged when nurse staffing was instead measured with the PES-NWI staffing and resource adequacy subscale (Supplemental Tables 2–3, SDC1, Supplemental Digital Content 1, https://links.lww.com/MLR/D242). Although prior research supports nurse-reported assessments as valid measures of features of the work environment, perception-based measures may differ from administrative staffing counts. Fewer nurse respondents were available in for-profit hospitals, in proportion to the smaller number and size of for-profit hospitals in the sample; any resulting imprecision in hospital-level staffing aggregates would generally attenuate rather than inflate mediated effects. Because the staffing measure and the nurse outcomes were drawn from the same survey, mediation estimates for nurse outcomes may be inflated by shared-source variance. The staffing mediator was aggregated to the hospital level while nurse outcomes were analyzed at the individual level, which attenuates this concern, and mediation was also substantial for patient outcomes and hospital ratings drawn from independent data sources (ie, Medicare claims and Hospital Compare).

The consistency of findings across patient, nurse, and hospital outcomes suggests that nurse staffing is a key, modifiable, and policy-relevant mechanism linking for-profit hospital ownership to performance. Workforce investments are not merely internal labor decisions but are closely tied to health care quality, patient safety, and the retention of a qualified nursing workforce.

## Supplementary Material
