# Supplementary material for: Patient Outcomes and Nurse Understaffing in For-Profit Hospitals
Source: Med Care. 2026 Sep 3;64(12):856–63. doi: 10.1097/MLR.0000000000002376 (PMC13742354; doi:10.1097/MLR.0000000000002376)
Supplement: Supplementary file 1 [file mlr-64-856-s001.docx]

**Supplemental Digital Content 1 (SDC1)**

Supplement to: Patient Outcomes and Nurse Understaffing in For-Profit Hospitals

**Contents**

**Supplemental Table 1.** Sensitivity of Nurse Staffing Mediation Effects to Unmeasured Mediator–Outcome Confounding

**Supplemental Table 2.** Causal Mediation Effects of Nurse Staffing Measured with the PES-NWI Staffing and Resource Adequacy Subscale on the Association Between For-Profit Hospital Status and Patient Outcomes

**Supplemental Table 3.** Causal Mediation Effects of Nurse Staffing Measured with the PES-NWI Staffing and Resource Adequacy Subscale on the Association Between For-Profit Hospital Status and Nurse Outcomes

| **Supplemental Table 1. Sensitivity of Nurse Staffing Mediation Effects to Unmeasured Mediator–Outcome Confounding** | |
| --- | --- |
| **Outcome** | **Critical ρ** |
| **Hospital Compare outcomes (hospital-level)** |  |
| HCAHPS overall hospital rating | 0.4 |
| Hybrid risk-standardized mortality | −0.3 |
| Hybrid risk-standardized readmission | −0.2 |
| **Nurse outcomes (hospital-level)** |  |
| Burnout | −0.5 |
| Intent to leave | −0.3 |
| Job dissatisfaction | −0.5 |
| Low confidence management will resolve safety issues | −0.6 |
| Quality of care rated poor or fair | −0.6 |
| Would not recommend hospital to family | −0.6 |
| Would not recommend as workplace | −0.6 |

ACME=average causal mediation effect. The critical ρ (rho) is the residual mediator–outcome correlation at which the unmeasured-confounder-adjusted ACME would equal zero; larger absolute values indicate greater robustness to unmeasured mediator–outcome confounding. Sensitivity analyses were conducted using the medsens procedure and were estimated on hospital-aggregated (single-level) data because medsens is not implemented for multilevel models. Sensitivity for the smaller-magnitude mediated effects (hybrid readmission) is weaker than for larger-magnitude effects, which is a mathematical property of sensitivity analyses rather than evidence that these effects are absent.

**Supplemental Table 2. Causal Mediation Effects of Nurse Staffing Measured with PES-NWI Staffing and Resource Adequacy Subscale on the Association Between For-Profit Hospital Status and Patient Outcomes**

|  |  |  |  |  |  |
| --- | --- | --- | --- | --- | --- |
| **Outcome** | **Total Effect, Estimate** | **Total Effect, 95% CI** | **ACME, Estimate** | **ACME, 95% CI** | **% Mediated** |
| **Surgical Patients** |  |  |  |  |  |
| 30-day Mortality | 0.48 | (0.24–0.73) | 0.26 | (0.17–0.37) | 53% |
| 30-day Readmission | 1.76 | (1.27–2.25) | 0.55 | (0.35–0.77) | 30% |
| **Medical Patients** |  |  |  |  |  |
| 30-day Mortality | 1.13 | (0.52–1.73) | 0.45 | (0.19–0.70) | 39% |
| 30-day Readmission | 2.04 | (1.54–2.53) | 0.38 | (0.18–0.59) | 18% |
| **Hospital-Level Outcomes** |  |  |  |  |  |
| Hybrid Mortality | 0.24 | (0.12–0.34) | 0.14 | (0.09–0.19) | 58% |
| Hybrid Readmission | 0.59 | (0.41–0.75) | 0.11 | (0.03–0.19) | 18% |
| Overall Hospital Rating | −4.44 | (−5.31–−3.61) | −1.65 | (−2.06–−1.26) | 37% |

ACME=average causal mediation effect; PES-NWI=Practice Environment Scale of the Nursing Work Index. Effects are reported as absolute differences in outcome probability for patient-level binary outcomes expressed in percentage points and as absolute differences in risk-standardized rates for hospital-level outcomes.

| **Supplemental Table 3. Causal Mediation Effects of Nurse Staffing Measured with PES-NWI Staffing and Resource Adequacy Subscale on the Association Between For-Profit Hospital Status and Nurse Outcomes** | | | | | |
| --- | --- | --- | --- | --- | --- |
| **Outcome** | **Total Effect, Estimate** | **Total Effect, 95% CI** | **ACME, Estimate** | **ACME, 95% CI** | **% Mediated** |
| Burnout | 9.3 | (6.0–12.6) | 8.7 | (7.1–10.5) | 94% |
| Intent to Leave | 11.9 | (9.0–15.2) | 5.1 | (3.8–6.8) | 44% |
| Job Dissatisfaction | 11.9 | (8.7–15.4) | 9.4 | (7.3–11.4) | 79% |
| Low confidence in management | 15.0 | (12.2–17.9) | 10.7 | (8.7–12.5) | 71% |
| Poor or fair quality of care | 17.1 | (12.8–20.9) | 14.3 | (11.5–17.3) | 84% |
| Would not recommend hospital to family | 22.8 | (18.3–27.3) | 15.6 | (12.3–19.1) | 68% |
| Would not recommend as workplace | 25.7 | (21.4–30.2) | 15.6 | (12.6–18.9) | 61% |

ACME=average causal mediation effect; PES-NWI=Practice Environment Scale of the Nursing Work Index. Effects are reported as absolute differences in outcome probability expressed in percentage points for nurse-level binary outcomes.
